# Impact of preoperative and constitutional alignment on soft tissue stiffness in robot-assisted total knee arthroplasty

**DOI:** 10.1186/s42836-025-00338-7

**Published:** 2025-10-14

**Authors:** Zhaolun Wang, Mingxue Chen, Yixin Zhou, Yunfeng Zhang, Dejin Yang, Chengshuai Zhang, Hao Tang, Yong Huang

**Affiliations:** 1 Department of Orthopaedic Surgery, Beijing Jishuitan Hospital, Capital Medical University, Beijing, 100035 China; 2 Fourth Clinical College of Peking University, Beijing, 100035 China

**Keywords:** Total knee arthroplasty, Soft tissue stiffness, Preoperative alignment, Constitutional alignment, Coronal Plane Alignment of the Knee (CPAK)

## Abstract

**Background:**

Although robot-assisted TKA improves alignment accuracy, the understanding of soft tissue stiffness remains limited. This study aimed to investigate the impact of preoperative and constitutional alignment on the stiffness of knee compartments during robot-assisted TKA.

**Methods:**

We included 151 patients who underwent primary robot-assisted TKA between May 2021 and May 2022. A digital joint-tensioning device was used intraoperatively to apply stepwise increasing tension (30–90 N) to the medial and lateral knee compartments. The device measured corresponding gap changes at 0°, 10°, and 90° of flexion. Linear regression was used to analyze the relationship between gap changes and applied tension, and the regression slope (K value) was used to compare stiffness between compartments. Preoperative factors, including hip-knee-ankle angle and Coronal Plane Alignment of the Knee (CPAK) subtypes, were assessed for their influence on stiffness.

**Results:**

There were significant differences in stiffness (K values) between medial and lateral compartments, particularly at higher flexion. The medial compartment generally showed greater stiffness. The medial-to-lateral stiffness ratio increased with greater varus alignment. Significant differences in K_M_/K_L_ ratios were found among CPAK subtypes at 0° and 10° flexion.

**Conclusion:**

This study introduced gap-tension regression for assessing soft tissue stiffness in robot-assisted TKA and showed that stiffness is influenced by preoperative and constitutional alignment. Varus alignment was associated with higher medial-to-lateral stiffness, and CPAK subtypes showed distinct stiffness patterns. These findings may help optimize soft tissue balancing and improve outcomes in TKA.

**Supplementary Information:**

The online version contains supplementary material available at 10.1186/s42836-025-00338-7.

## Introduction

The success of total knee arthroplasty (TKA) depends on the harmonious interaction between the newly implanted prosthesis and the surrounding soft tissue envelope. Poor soft tissue balancing can result in conflict between implant surface-guided and soft tissue-guided knee motion, potentially leading to severe complications such as instability and premature polyethylene wear [1–3].

Unfortunately, despite advancements in robot-assisted TKA, the definition of soft tissue balance remains elusive and controversial, posing significant challenges to improving patient outcomes [4]. Traditional soft tissue balancing aims to create equal, symmetrical extension and flexion gaps [5]. Surgeons achieve this through soft tissue release, bone cuts, or a combination of both. Regardless of the technique used, the procedure is highly subjective and experience-dependent [6]. Over the past two decades, researchers have attempted to quantify soft tissue balance using various methods, including pressure sensor inserts [4, 7–10] and tensor-based methods [11–14]. Notably, Wasielewski et al. introduced a smart tibial insert that recorded pressures at the tibiofemoral interface, linking these pressures to the condylar liftoff observed during postoperative fluoroscopy [15]. In a multicenter study, Gustke et al. defined balanced TKA as having a mediolateral intercompartmental pressure difference (ICPD), measured using smart tibial inserts of less than 15 lb (66.7 N) at all flexion angles [4]. Conversely, Wakelin et al. quantified intraoperative soft tissue balance by applying predetermined tension to the medial and lateral compartments and defined soft tissue balance based on the resulting gap difference, which was significantly correlated with postoperative functional outcomes [12].

However, the previously mentioned studies only measured gap values at a fixed tension or measured tension at a fixed gap value (insert thickness), and neither of them has gauged soft tissue stiffness, that is, the resistance of soft tissue to different tensions. Therefore, this study aimed to present a method for assessing soft tissue stiffness in TKA using a novel device that applies stepwise increasing tension to the medial and lateral compartments to measure the corresponding gap changes. The primary objectives are: 1) to describe and quantify soft tissue stiffness in TKA; 2) to compare the differences in stiffness between the medial and lateral compartments; and 3) to explore factors affecting soft tissue stiffness, such as preoperative alignment, constitutional alignment, and prosthesis type.

## Patients and methods

### Patient selection

With the approval of the Institutional Review Board, patients who underwent primary robotic-assisted TKA and intraoperative measurements using a digital joint-tensioning device between May 2021 and May 2022 were included in this study. All surgeries were performed by the same experienced surgeon (YZ). The exclusion criteria were (1) inflammatory arthritis, (2) revision TKA, and (3) incomplete intraoperative soft-tissue measurement data. Finally, 151 patients were included in this study. The patient demographics are shown in Table 1.

**Table 1:** Patient demographics and preoperative characteristics

Characteristic	Data
Age (years)	67.7 ± 6.5
Female sex	120 (79.5%)
Body mass index (kg/m^2^)	27.2 ± 3.6
HKA (°)	172.9 ± 6.5
LDFA (°)	88.4 ± 2.9
MPTA (°)	84.8 ± 3.7
aHKA (°)	− 3.7 ± 5.4
JLO (°)	173.2 ± 3.9
CR prosthesis	116 (76.8%)

### Operative technique

**Fig. 1:**
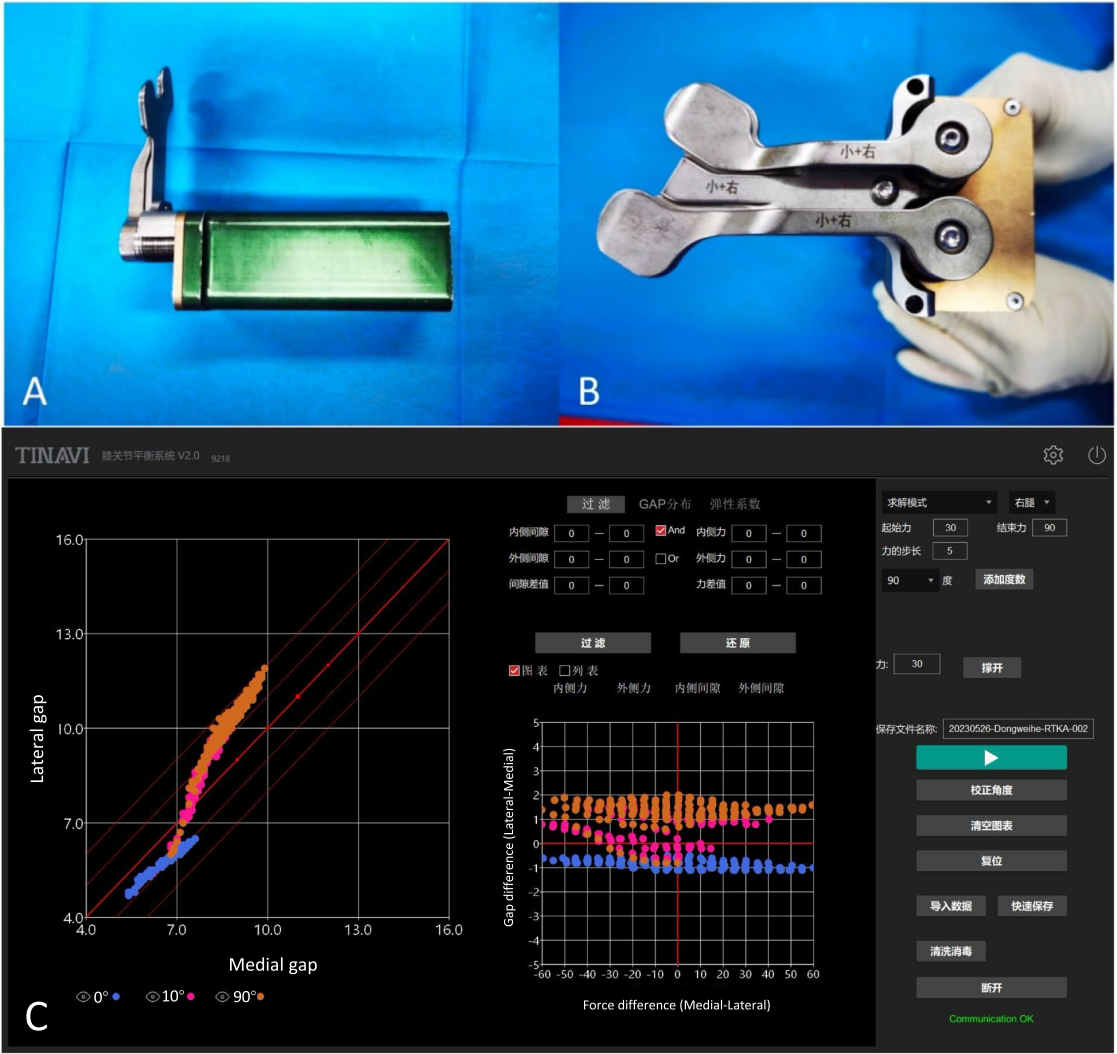
**A** Latertal view of the digital tensor (Tinavi Corp., Beijing, China) used in this study; (**B**) Top view of the digital tensor; (**C**) Display interface showing two matrices: the left matrix presents the medial and lateral gap measurements, while the right matrix displays the differences in medial–lateral force and gap, with different colors representing measurements at various flexion angles

All procedures were performed using the Mako Robotic-assisted Total Knee System (Stryker Corp, Mahwah, NJ, USA), employing either a cruciate-retaining (CR) or posterior-stabilized (PS) prosthesis (Triathlon, Stryker Corp). After medial parapatellar arthrotomy, the accessible osteophytes were removed. Following bone registration, a varus/valgus stress test was conducted with the joint in extension, and a spoon was inserted at 90° flexion to evaluate the maximum gap width. Based on the pre-resection assessment, the component position was adjusted to balance the extension and flexion gaps, ensuring that the lower limb alignment remained within a 3° deviation from the neutral mechanical alignment. After trial implant insertion but prior to any soft tissue release, a digital tensor (Tinavi Corp., Beijing, China) was inserted (Fig. 1). The tensor applied varying tension from 30 to 90 N in 5 N increments, independently to the medial and lateral compartments, enabling the measurement of gap changes for each unique combination. The measurement accuracy of gaps and tensions was ± 0.6 mm and ± 1.5N, respectively [16]. Measurements were performed with the patella reduced at 0° (full extension), 10° (early flexion with the posterior capsule relaxed), and 90° of knee flexion. During this process, the heel was gently supported by the surgeon’s hand, whereas an experienced assistant held the distal femur with both hands and applied a steady upward force sufficient to balance the weight of the leg. This technique ensured that the knee was in a passive, gravity-neutralized state during stiffness measurements, minimizing external forces that could confound the results.

### Preoperative radiographic evaluation

Preoperative weight-bearing full-length radiographs of the lower extremities were imported into Mimics software (version 21.0) for measurement, with a resolution of 0.01 mm and 0.01° for length and angle, respectively. The following parameters were measured: (1) hip-knee-ankle angle (HKA), the medial angle between the tibial and femoral mechanical axes; (2) mechanical lateral distal femoral angle (mLDFA), lateral angle between the femoral mechanical axis and the distal femoral joint line; (3) medial proximal tibial angle (MPTA), the medial angle between the tibial mechanical axis and the proximal tibial joint line. The arithmetic HKA (aHKA) and joint line obliquity (JLO) were calculated using the following algorithms: *aHKA* = *MPTA – mLDFA; JLO* = *MPTA* + *mLDFA*. The patients were subsequently classified into different Coronal Plane Alignment of the Knee (CPAK) subtypes based on their aHKA and JLO [17].

### Data analysis

Gap values with equal tension at each flexion angle were extracted to simplify the model and ensure consistency and comparability. A simple linear regression model was used to fit the relationship between the gap and tension measurements of the medial and lateral compartments at different flexion angles. The coefficient of determination (R^2^), regression slope (K, N/mm), and intercept term (β) were calculated. The K values of the medial and lateral compartments were compared using a matched *t*-test. The K values and their ratios (K_M_/K_L_) at different flexion angles were compared among the CPAK subtypes, preoperative HKA groups, CR/PS groups, and sex groups using a one-way ANOVA or two-sample *t*-test. The correlations of K_M_/K_L_ with preoperative HKA and BMI were analyzed using Pearson’s correlation coefficient.

## Results

**Fig. 2:**
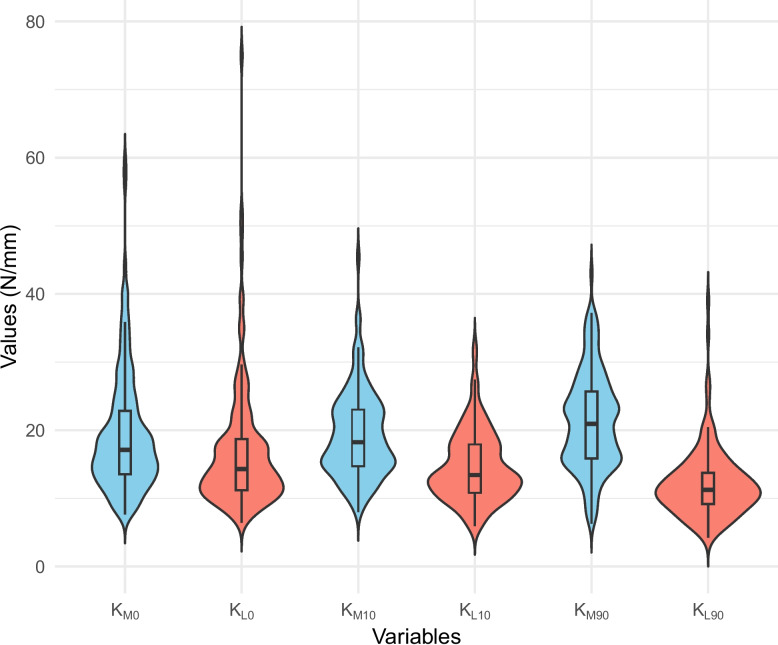
Violin plot of medial and lateral K values and different flexion angles. The width of each violin shape represents the data density at different y-values, estimated using Kernel Density Estimation (KDE). Inside each violin, a boxplot shows the median (thick black line), interquartile range (IQR, black box), and whiskers extending to the minimum and maximum values within 1.5 × IQR

The gap-tension relationship of the medial and lateral compartments was well fitted by a linear regression model (R^2^ > 0.9). K values of the medial and lateral compartments increased with flexion, and the medial compartment remained stiffer at all flexion angles (Table 2; Fig. 2). The ratio of the medial and lateral compartment K values (K_M_/K_L_) increased with the degree of flexion.

**Table 2:** Stiffness (K value) of medial and lateral compartments at different flexion angles

	**K value**			**Intercept**			**R**^**2**^	
**Flexion angle**	**Medial**	**Lateral**	***P* value**	**Medial**	**Lateral**	***P* value**	**Medial**	**Lateral**
0°	19.40 ± 8.92	16.83 ± 9.31	< 0.001	− 130.62 ± 88.9	− 108.92 ± 79.36	< 0.001	0.94 ± 0.12	0.93 ± 0.12
10°	19.17 ± 6.35	14.57 ± 5.11	< 0.001	− 156.90 ± 84.38	− 116.27 ± 70.87	< 0.001	0.95 ± 0.07	0.93 ± 0.13
90°	21.24 ± 7.13	12.08 ± 5.04	< 0.001	− 160.76 ± 74.83	− 82.16 ± 58.91	< 0.001	0.94 ± 0.13	0.91 ± 0.13

**Fig. 3:**
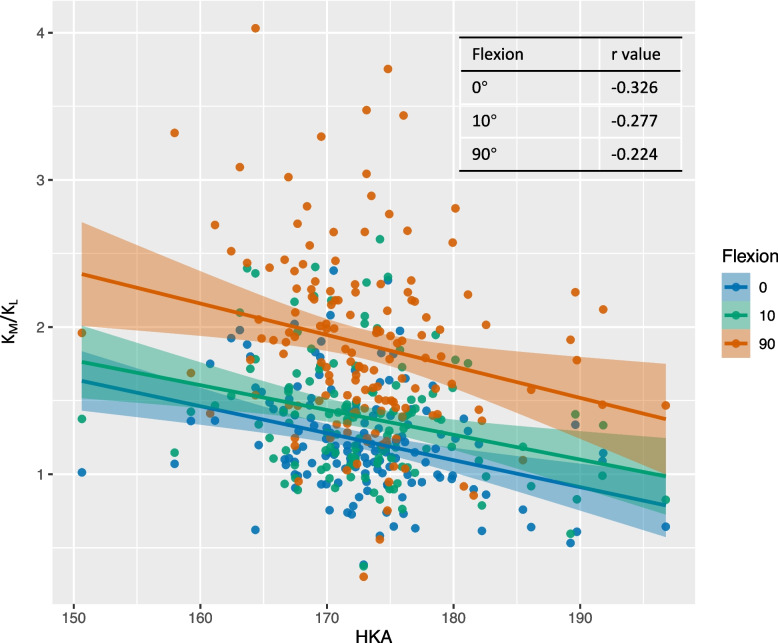
Relationship between preoperative hip-knee-ankle (HKA) angle and medial to lateral compartment stiffness ratio (K_M_/K_L_) at different flexion angles

**Fig. 4:**
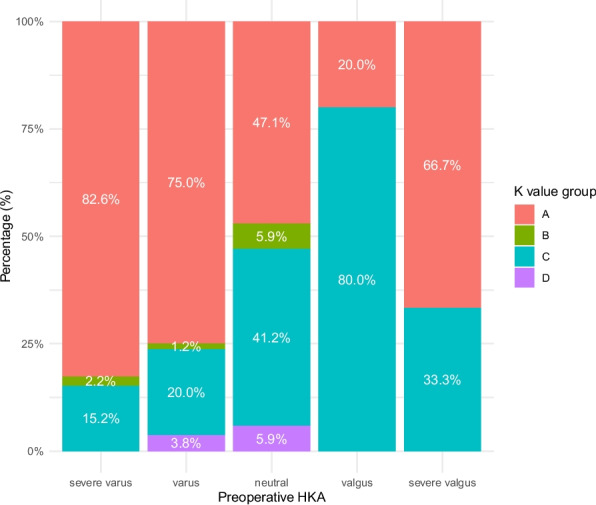
Proportion of patients with different combinations of medial and lateral K values. A: K_M0_ > K_L0_, K_M90_ > K_L90_, B: K_M0_ > K_L0_, K_M90_ < K_L90_, C: K_M0_ < K_L0_, K_M90_ > K_L90_, D: K_M0_ < K_L0_, K_M90_ < K_L90_

Preoperative HKA significantly affected K_M_/K_L_, which increased with varus deformity (Table 3). The Pearson correlation coefficients between the preoperative HKA and K_M_/K_L_ were − 0.326, − 0.277, and − 0.224 at 0°, 10°, and 90° of flexion, respectively (Fig. 3). In full extension, K_M_/K_L_ was greater than 1.0 in patients with neutral or varus alignment and less than 1.0 in patients with valgus alignment. However, at 90° of flexion, K_M_/K_L_ was greater than 1.5 in all groups, including those with severe valgus deformity. As the degree of varus alignment increased, the proportion of patients with K_M_ values greater than K_L_ values also increased (Fig. 4). However, for patients with varus alignment, 23.8% still had greater K_L_ than K_M_ in extension, and 5.0% had greater K_L_ than K_M_ at 90° of flexion. Among patients with severe varus alignment, 17.4% had greater K_L_ than K_M_ extension.

**Table 3:** Effect of preoperative hip-knee-ankle (HKA) alignment on medial and lateral compartment stiffness ratios (K_m_/K_l_)

Flexion angle	HKA	< 170°	170° ~ 177°	177° ~ 183°	183° ~ 190°	> 190°	*P* value
		**(*N* = 46)**	**(*N* = 80)**	**(*N* = 17)**	**(*N* = 5)**	**(*N* = 3)**	
0°	K_M_	20.08 ± 7.84	18.88 ± 8.72	18.76 ± 6.66	19.36 ± 12.29	26.34 ± 28.55	0.661
	K_L_	15.39 ± 7.17	16.69 ± 9.81	17.82 ± 8.53	23.78 ± 8.36	25.14 ± 23.18	0.164
	K_M_/K_L_	1.37 ± 0.34	1.20 ± 0.37	1.11 ± 0.24	0.78 ± 0.32	0.96 ± 0.27	< 0.001
10°	K_M_	19.52 ± 5.24	18.5 ± 6.29	22.65 ± 7.14	18.13 ± 11.12	16.41 ± 5.54	0.246
	K_L_	13.39 ± 4.12	14.45 ± 5.16	16.86 ± 5.72	18.07 ± 8.12	15.61 ± 3.27	0.133
	K_M_/K_L_	1.53 ± 0.43	1.35 ± 0.4	1.39 ± 0.34	0.99 ± 0.32	1.05 ± 0.26	0.017
90°	K_M_	21.52 ± 6.52	21.29 ± 7.42	21.09 ± 7.95	20.31 ± 6.8	18.01 ± 8.06	0.943
	K_L_	10.65 ± 3.81	12.81 ± 5.5	12.53 ± 5.07	12.71 ± 6.11	10.79 ± 5.23	0.226
	K_M_/K_L_	2.14 ± 0.61	1.78 ± 0.63	1.75 ± 0.52	1.72 ± 0.42	1.69 ± 0.38	0.025

Significant differences were observed between the mean K_M_/K_L_ of the different CPAK subgroups at 0° and 10° of flexion (Table 4). More specifically, the K_M_/K_L_ values tended to be higher in CPAK I, II, and IV groups. The presence or absence of the PCL had no significant effect on the medial and lateral K values or their ratios, except for K_M_/K_L_ at 10° of flexion (Table 5).

**Table 4:** Comparison of stiffness (K value) among different coronal plane alignment of the knee (CPAK) subgroups

Flexion angle	CPAK	I	II	III	IV	V	*P* value
		**(*N* = 88)**	**(*N* = 30)**	**(*N* = 15)**	**(*N* = 15)**	**(*N* = 3)**	
0°	K_M_	18.92 ± 8.24	19.46 ± 8.2	20.91 ± 14.19	20.44 ± 8.62	15.22 ± 3.28	0.828
	K_L_	16.05 ± 9.87	16.6 ± 7.19	23.48 ± 12.43	15.62 ± 4.77	16.18 ± 2.47	0.090
	K_M_/K_L_	1.27 ± 0.35	1.2 ± 0.3	0.88 ± 0.28	1.33 ± 0.44	0.97 ± 0.34	0.001
10°	K_M_	18.51 ± 5.79	21.39 ± 7.15	18.13 ± 7.26	18.74 ± 6.32	24.35 ± 8.74	0.239
	K_L_	13.62 ± 4.8	15.85 ± 5.41	17.07 ± 5.86	14.11 ± 4.4	18.74 ± 3.34	0.066
	K_M_/K_L_	1.45 ± 0.45	1.39 ± 0.33	1.08 ± 0.29	1.37 ± 0.4	1.28 ± 0.24	0.048
90°	K_M_	21.02 ± 7.31	20.31 ± 6.04	23.91 ± 8.76	21.52 ± 7.15	22.95 ± 3.07	0.608
	K_L_	11.61 ± 4.26	12.39 ± 6	14.79 ± 8.03	11.19 ± 2.91	13.93 ± 3.65	0.220
	K_M_/K_L_	1.93 ± 0.66	1.79 ± 0.61	1.75 ± 0.44	1.95 ± 0.58	1.69 ± 0.25	0.666

CPAK subtypes were defined based on joint line orientation (JLO) and arithmetic hip-knee-ankle angle (aHKA): Type I and IV represent constitutional varus (aHKA < –2°), Types II and V represent constitutional neutral (aHKA –2° to 2°), and Type III represents constitutional valgus (aHKA > 2°). Types I, II, and III had JLO < 177°, while Types IV and V had JLO between 177° and 183°

**Table 5:** Influence of prosthesis type (cruciate retaining vs. posterior stabilized) on medial and lateral compartment stiffness

Flexion angle	Prosthesis	CR	PS	*P* value
		***N* = 116**	***N* = 35**	
0°	K_M_	19.99 ± 9.10	17.41 ± 8.05	0.117
	K_L_	17.49 ± 9.81	14.57 ± 6.99	0.056
	K_M_/K_L_	1.23 ± 0.38	1.23 ± 0.33	0.961
10°	K_M_	18.85 ± 6.14	20.21 ± 7.03	0.357
	K_L_	14.88 ± 5.29	13.57 ± 4.42	0.190
	K_M_/K_L_	1.34 ± 0.41	1.54 ± 0.39	0.021
90°	K_M_	21.31 ± 7.39	21.00 ± 6.30	0.807
	K_L_	12.25 ± 5.43	11.48 ± 3.42	0.322
	K_M_/K_L_	1.88 ± 0.64	1.91 ± 0.56	0.740

Overall, sex and BMI showed minimal influence on compartmental stiffness (Supplementary Tables S1 and S2). Females demonstrated slightly higher lateral stiffness at 0° (*P* = 0.038) and a higher K_M_/K_L_ ratio at 90° (*P* = 0.023), while no other sex-related differences were identified. BMI was not significantly associated with medial stiffness, lateral stiffness, or stiffness ratios across all flexion angles (all *P* > 0.05).

## Discussion

In the past two decades, researchers have attempted to establish objective methods for quantifying soft tissue balance, including those based on smart tibial inserts [4] and joint-tensioning devices [11, 12]. However, these methods raise new questions regarding whether soft tissue balance should be defined by gap or tension, causing confusion among surgeons. More importantly, these studies typically provided only single-point measurements of tension or gap, without elucidating the intrinsic relationship between them, namely, soft tissue stiffness.

In our study, we used a robot-assisted TKA cohort and a novel joint-tensioning device that gauged soft-tissue stiffness based on stiffness curves. Previous studies using joint tensioning devices measured gap widths with a predetermined fixed tension [12, 18]. They used the resulting gap differences between the medial and lateral compartments to define coronal balance [19]. However, this balance does not necessarily translate into pressure balance after prosthesis implantation, particularly if the thickness of the implant does not match the measured gap. As reported in this study, due to the different K values of the medial and lateral compartments, a simultaneous increase in the medial and lateral gaps would result in different force changes.

Similar to our approach, Asano et al. [20] and Heesterbeek et al. [21] also evaluated soft tissue stiffness by applying variable distraction forces and constructing force–displacement curves. Both studies reported a non-linear relationship between joint gap and tension, typically consisting of an initial low-stiffness region (toe phase) followed by a stiffer linear region (elastic phase). Heesterbeek et al. further identified the transition point between these two phases and suggested it as the appropriate tension level for gap balancing [21]. However, unlike our study, their measurement setup did not eliminate the effect of femoral weight. Moreover, subsequent studies using smart tibial inserts have demonstrated that postoperative soft tissue tensions in TKA are considerably higher than the transition point reported by Heesterbeek et al. (instead lying within the elastic phase) [8, 9]. Therefore, in our study, by applying a moderate level of distraction force, the majority of stiffness curves were satisfactorily approximated by a single linear fit (mean R^2^ > 0.9), and a clear non-linear transition was not observed, because characterizing the elastic behavior of soft tissues within this clinically relevant range is more applicable for intraoperative decision-making. In addition, neither Asano et al. nor Heesterbeek et al. explored the factors influencing the stiffness coefficient in the elastic phase, which was the focus of our investigation.

Overall, the mean K value of the medial compartment is larger than that of the lateral compartment, indicating that the medial soft tissue is “stiffer”. This difference was more pronounced at 90° of flexion than at full extension. Understanding this difference is crucial for achieving soft tissue balance during TKA. Due to this disparity, equalizing the medial and lateral gaps under specific tension does not guarantee a pressure balance after prosthesis implantation. Furthermore, simultaneous increases or decreases in the medial and lateral gaps result in different changes in tension in the medial and lateral compartments, implying that simply adjusting the thickness of the tibial insert may lead to an increased ICPD. Knowledge of the gap-tension curves of the medial and lateral compartments allows for predictable adjustments to the ICPD by changing the direction of the bone cuts. For example, the internal rotation of the osteotomy line around the most distal point of the lateral femoral condyle changes the medial gap.

1 $$\Delta h=sin\theta \times W$$

where *W* denotes the mediolateral width. The resulting change in medial compartment pressure can be calculated as

2 $$\Delta {F}_{M}={K}_{M}\times sin\theta \times W$$

Generally, a 1-degree change in coronal osteotomy of the distal femur results in a 1 mm gap difference on one side [22]. However, the force change due to this gap variation depends on soft tissue stiffness, that is, the K value. Therefore, Eq. (2) allows estimation of the medial force increase after femoral rotation.

The differences in the medial and lateral elasticities were likely related to the kinematics of the native knee. In a native knee, there is a progressive posterior translation of the lateral femoral condyle on the tibial plateau during flexion, known as “posterior femoral rollback”, whereas the accompanying posterior translation of the medial femoral condyle is negligible and referred to as “medial pivoting” of the knee [23]. Many surgeons have attempted to reproduce this mechanism by altering the geometry of prostheses, such as using medial-pivoting prostheses. However, they overlook the important fact that the motion of the knee joint is guided not only by the geometry of the prosthesis but also by the stiffness of the surrounding soft tissue. Our study demonstrated that differences in the elastic behaviors of the medial and lateral soft tissues may affect this mechanism, as the K values of the lateral compartment tended to decrease from 0° to 90°, whereas the K values of the medial compartment did not change significantly. Reproducing natural knee kinematics requires harmony between soft-tissue stiffness and prosthesis geometry. Failure to understand and respect soft tissue elastic behavior may lead to kinematic conflict between soft tissue-guided motion and implant geometry-guided motion. This may explain why some patients are unable to regain native knee kinematics even with MP prostheses [24].

We explored the factors affecting the ratio of medial to lateral compartment K values (K_M_/K_L_), including the presence or absence of the posterior cruciate ligament, preoperative HKA, and CPAK classification. The preoperative HKA alignment was the most influential factor, with K_M_/K_L_ increasing with the severity of the varus deformity. This is likely due to stiffness changes in the lateral compartment, as the K value of the lateral compartment decreased by approximately 40% from the most valgus to the most varus group. In addition, K_M_/K_L_ increased with increasing flexion angle in the same patient. For patients with severe varus deformity, the K_M_/K_L_ was approximately 1.5 in full extension and more than 2 at 90° of flexion, making soft tissue balance challenging without soft tissue release. This is consistent with previous findings [25]. Ushio et al. compared the differences in medial and lateral soft tissue stiffness in osteoarthritic knees using varus/valgus stress radiographs and found that varus deformity correlated significantly with lateral laxity [25]. In individuals with severe varus deformities, the lateral soft tissues remodel due to stretching caused by lateral thrusting movements during daily weight-bearing activities [26]. Concurrently, contracture of the posterior joint capsule partially counteracts this effect in the extended position, resulting in the difference between medial and lateral stiffness becoming more pronounced in the flexion position [27].

Despite the association of stiffness curves with preoperative alignment, we also found some outliers reflecting large individual differences in soft tissue stiffness. For example, 23.8% of patients with preoperative varus alignment had greater K_L_ than K_M_ in extension, while 5.0% had greater K_L_ than K_M_ at 90° of flexion. For these patients, intraoperative medial soft tissue release should be considered with caution, as it may increase the stiffness difference between the medial and lateral soft tissues.

Another important finding of this study is that the CPAK classification also significantly affects the medial/lateral stiffness differences. Generally, constitutional varus alignment led to greater K_M_/K_L_ ratios, while constitutional valgus alignment led to smaller K_M_/K_L_ ratios. Compared to the neutral JLO, an apex distal JLO may also lead to larger K_M_/K_L_ ratios. It is worth noting that the above findings were limited to the extension or 10° flexion positions; no statistically significant differences were observed at 90° flexion between CPAK groups. This may explain why there was an increased risk of imbalance between medial and lateral pressures in patients with CPAK types I, II, and IV in the study by MacDessi et al. [17]. To our knowledge, no previous study has reported the influence of constitutional alignment on the individual soft tissue stiffness. Our findings suggested that a patient’s constitutional soft tissue stiffness may be adapted to his constitutional alignment and may gradually change with the alignment changes due to the progression of arthritis. This suggests significant inter-individual variability in soft tissue stiffness within the OA population.

This study had some limitations. First, it proposed a new method using stiffness curves to describe soft tissue balance, but did not correlate intraoperative measurements with follow-up data. Future studies could explore the correlation of these parameters with postoperative functional scores and prosthesis survival. Second, the limited number of valgus deformity cases may restrict the applicability of our conclusions to valgus knees. In conclusion, this study presented a new method for describing the elastic behavior of knee soft tissues and found that the medial and lateral compartments exhibit different elastic behaviors, which increase with knee flexion angle and preoperative varus deformity. Future studies should establish quantitative soft tissue balance strategies based on these findings.

## Supplementary Information

Supplementary Material 1. Table S1. Effect of Sex on Medial and Lateral Compartment Stiffness, Table S2. Pearson’s correlation coefficients of BMI on Medial and Lateral Compartment Stiffness.

## Data Availability

No datasets were generated or analysed during the current study.

## Abbreviation

TKA: Total Knee Arthroplasty
CPAK: Coronal Plane Alignment of the Knee
CR: Cruciate-Retaining
PS: Posterior-Stabilized
HKA: Hip-Knee-Ankle Angle
MPTA: Medial Proximal Tibial Angle
mLDFA: Mechanical Lateral Distal Femoral Angle
aHKA: Arithmetic Hip-Knee-Ankle Angle
JLO: Joint Line Obliquity
K_L_: Lateral Stiffness (K value of lateral compartment)
K_M_: Medial Stiffness (K value of medial compartment)
ICPD: Intercompartmental Pressure Difference
