# Supplementary material for: Impact of preoperative and constitutional alignment on soft tissue stiffness in robot-assisted total knee arthroplasty
Source: Arthroplasty. 2025 Oct 14;7:53. doi: 10.1186/s42836-025-00338-7 (PMC12519729; doi:10.1186/s42836-025-00338-7)
Supplement: Supplementary file 1 — Supplementary Material 1. Table S1. Effect of Sex on Medial and Lateral Compartment Stiffness, Table S2. Pearson’s correlation coefficients of BMI on Medial and Lateral Compartment Stiffness. [file 42836_2025_338_MOESM1_ESM.docx]

Supplementary table 1 Effect of Sex on Medial and Lateral Compartment Stiffness

| Flexion angle | Stiffness | Female | Male | P value |
| --- | --- | --- | --- | --- |
| 0° | K_M_ | 19.84 ± 9.37 | 17.26 ± 6.27 | 0.074 |
|  | K_L_ | 17.40 ± 10.08 | 14.62 ± 5.25 | 0.038 |
|  | K_M_/K_L_ | 1.22 ± 0.38 | 1.21 ± 0.28 | 0.883 |
| 10° | K_M_ | 19.13 ± 6.41 | 19.32 ± 6.39 | 0.899 |
|  | K_L_ | 14.69 ± 5.22 | 14.26 ± 4.80 | 0.695 |
|  | K_M_/K_L_ | 1.38 ± 0.44 | 1.39 ± 0.30 | 0.948 |
| 90° | K_M_ | 21.43 ± 7.38 | 20.50 ± 6.10 | 0.481 |
|  | K_L_ | 12.05 ± 5.37 | 12.17 ± 3.51 | 0.885 |
|  | K_M_/K_L_ | 1.93 ± 0.66 | 1.72 ± 0.37 | 0.023 |

Supplementary table 2 Pearson’s correlation coefficients of BMI on Medial and Lateral Compartment Stiffness

| Flexion angle | Stiffness | Pearson’s coefficient | P value |
| --- | --- | --- | --- |
| 0° | K_M_ | 0.11 | 0.189 |
|  | K_L_ | 0.01 | 0.922 |
|  | K_M_/K_L_ | 0.16 | 0.051 |
| 10° | K_M_ | -0.13 | 0.153 |
|  | K_L_ | -0.11 | 0.239 |
|  | K_M_/K_L_ | -0.03 | 0.752 |
| 90° | K_M_ | 0.05 | 0.524 |
|  | K_L_ | -0.01 | 0.859 |
|  | K_M_/K_L_ | 0.06 | 0.496 |
